# Percentage of mean arterial pressure as a marker of atherosclerosis for detecting patients with coronary artery disease

**DOI:** 10.1038/s41440-023-01442-4

**Published:** 2023-10-04

**Authors:** Tatsuya Maruhashi, Masato Kajikawa, Shinji Kishimoto, Takayuki Yamaji, Takahiro Harada, Yu Hashimoto, Aya Mizobuchi, Shunsuke Tanigawa, Farina Mohamad Yusoff, Yukiko Nakano, Kazuaki Chayama, Ayumu Nakashima, Chikara Goto, Yukihito Higashi

**Affiliations:** 1 Department of Regenerative Medicine, Research Institute for Radiation Biology and Medicine, Hiroshima University, 1-2-3 Kasumi, Minami-ku, Hiroshima, Japan; 2 Division of Regeneration and Medicine, Medical Center for Translational and Clinical Research, Hiroshima University Hospital, 1-2-3 Kasumi, Minami-ku, Hiroshima, Japan; 3 Department of Cardiovascular Medicine, Graduate School of Biomedical and Health Sciences, Hiroshima University, 1-2-3 Kasumi, Minami-ku, Hiroshima, Japan; 4 Department of Medicine and Molecular Science, Hiroshima University Graduate School of Biomedical Sciences, Hiroshima University, 1-2-3 Kasumi, Minami-ku, Hiroshima, Japan; 5 Department of Stem Cell Biology and Medicine, Graduate School of Biomedical and Sciences, Hiroshima University, 1-2-3 Kasumi, Minami-ku, Hiroshima, Japan; 6 Department of Rehabilitation, Faculty of general Rehabilitation, Hiroshima International University, 555-36 Kurosegakuendai, Higashihiroshima, Japan

**Keywords:** Ankle-brachial index, Coronary artery disease, Percentage of mean arterial pressure, Pulse volume recording

## Abstract

**Abstract:**

The percentage of mean arterial pressure (%MAP) is the height of the mean arterial waveform divided by the peak amplitude of the waveform of pulse volume recording. The purpose of this study was to determine whether the cutoff value of 45% for %MAP at the ankle, which is recommended for the diagnosis of lower extremity artery disease, in combination with ankle-brachial index (ABI) is useful for detecting patients with clinical coronary artery disease (CAD) and investigate the optimal cutoff value of %MAP to diagnose patients with CAD.　We measured ABI and %MAP in 2213 subjects　(mean age: 61.2 ± 15.5 years). Multivariate analysis revealed that %MAP ≥ 45% was significantly associated with a higher risk of CAD after adjusting for traditional cardiovascular risk factors (odds ratio [OR], 2.14; 95% confidence interval [CI], 1.43–3.21; *p* < 0.001). However, the association was no longer significant after adjusting for ABI (OR, 1.39; 95% CI, 0.83–2.33; *p* = 0.21), whereas ABI was significantly associated with CAD (OR, 0.98; 95% CI, 0.97–0.99; *p* = 0.005). The optimal cutoff value of %MAP derived from a receiver operating characteristic curve to diagnose CAD was 40.3%. Multivariate analysis revealed that %MAP ≥ 40.3% was significantly associated with a higher risk of CAD (OR, 1.63; 95% CI, 1.19–2.24; *p* = 0.002) independent of ABI (OR, 0.98; 95% CI, 0.97–0.99; *p* = 0.002). The cutoff value of 40.3%, but not 45%, for %MAP may be useful for detecting patients with advanced atherosclerosis and for cardiovascular risk assessment independent of ABI.

**Registration Information:**

http://www.umin.ac.jp (University Hospital Medical Information Network Clinical Trials Registry) (UMIN000039512)

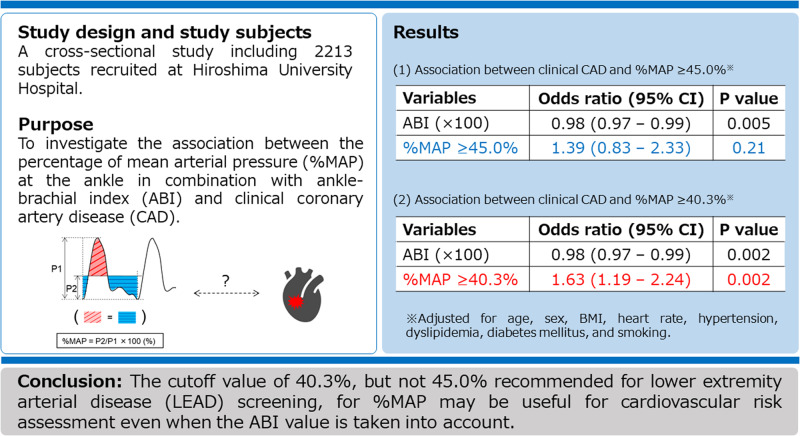

## Introduction

Measurement of ankle-brachial index (ABI), the ratio of ankle systolic blood pressure to brachial systolic blood pressure, has been widely used as a noninvasive screening method for detecting patients with lower extremity artery disease (LEAD). ABI is not only an indicator of LEAD but also an indicator of systemic atherosclerosis and, therefore, can serve as a vascular marker of atherosclerosis for cardiovascular risk assessment. Indeed, several population-based cohort studies have shown that a lower ABI is associated with higher rates of concomitant cardiovascular disease and higher incidence of cardiovascular events [1–6]. Therefore, patients with a low ABI value should be regarded as being at high cardiovascular risk.

In addition to ABI, waveforms of pulse volume recording at the ankle obtained by using a plethysmographic technique can be used for noninvasive detection of LEAD [7–9]. Recent advancements in oscillometric cuff technology have made it possible to simultaneously obtain accurate waveforms of pulse volume recording at the ankle in a short time when measuring ABI by using an automated oscillometric device, which can lead to clinical application of pulse volume recording parameters calculated from high-quality pulse volume waveforms. Percentage of mean arterial pressure (%MAP), one of the parameters automatically calculated from pulse volume waveforms, is the height of the mean area of the arterial waveform divided by the peak amplitude [10]. In patients with hemodynamically occlusive lesions in the lower extremity artery, the pulse volume waveform at the ankle tends to be blunted and, consequently, %MAP should increase. Indeed, the results of a clinical study have shown that %MAP at the ankle increases with increasing stenosis severity in the lower extremity artery and that the cutoff value of 45% for %MAP is proposed for the diagnosis of LEAD [10–12]. These findings indicate the possibility that %MAP, as well as ABI, can be used as a vascular marker for detecting patients with advanced atherosclerosis. However, there is little information on the usefulness of %MAP alone or in combination with ABI for cardiovascular risk assessment. Therefore, we investigated the association between %MAP and clinical coronary artery disease (CAD) to determine whether the cutoff value of 45% for %MAP is useful for detecting patients at high cardiovascular risk in a large number of well-characterized subjects. In addition, we investigated the optimal cutoff value of %MAP to diagnose clinical CAD.

Point of View

- **Clinical relevance**
  The percentage of mean arterial pressure (%MAP), one of the parameters automatically calculated from pulse volume wave forms, at the ankle may be useful for detecting patients with advanced atherosclerosis even when the ankle-brachial index (ABI) value is taken into account.
- **Future direction**
  Further studies are needed to determine whether %MAP can serve as a prognostic marker of cardiovascular events independent of ABI.
- **Consideration for the Asian population**
  The device used for the measurements of ABI and %MAP in this study is widely adopted in Asia, especially in East Asia, but not in Western countries.

## Methods

Data that support the findings of this study are available from the corresponding author on reasonable request.

### Subjects

This study was a cross-sectional study. Between January 2008 and December 2019, a total of 2749 subjects were recruited for measurements of ABI and pulse volume recording for calculation of %MAP from participants who visited the outpatient cardiology clinic or who underwent health-screening examinations at Hiroshima University Hospital. Some of the data have been previously reported elsewhere [13, 14]. Participants with severe aortic stenosis or aortic regurgitation (*n* = 35), atrial fibrillation (*n* = 183), LEAD defined as critical limb ischemia (*n* = 56), a history of major amputation (*n* = 55) or minor amputation (*n* = 12) or previous intervention including angioplasty or bypass graft (*n* = 76), and those with missing information on a history of CAD (*n* = 23) were excluded. Participants with an ABI ≥ 1.4 (*n* = 96) were further excluded. Finally, 2213 participants (1361 men and 852 women; mean age: 61.2 ± 15.5 years) with an ABI < 1.4 were enrolled in this study. Hypertension was defined as treatment with oral antihypertensive drugs or systolic blood pressure of more than 140 mmHg and/or diastolic blood pressure of more than 90 mmHg measured in a sitting position on at least three different occasions without medication [15]. Diabetes was defined according to the American Diabetes Association recommendation [16]. Dyslipidemia was defined according to the third report of the National Cholesterol Education Program [17]. Smokers were defined as those who had ever smoked. CAD was defined as a history of myocardial infarction and/or angina pectoris. Myocardial infarction was defined as organic occlusion of at least 1 coronary artery confirmed by coronary angiography (CAG) with or without a history of a coronary revascularization procedure including percutaneous coronary intervention and/or coronary artery bypass grafting. Angina pectoris was defined as organic stenosis (≥50%) of at least one coronary artery confirmed by CAG and a history of chest pain with or without a history of a coronary revascularization procedure. The vascular tests were performed without withholding medications. The ethical committees of our institutions (Hiroshima University Hospital institutional review board) approved the study protocol. Written informed consent for participation in the study was obtained from all subjects.

### Study protocol

The subjects fasted the previous night for at least 8 h and abstained from consuming alcohol and caffeine and from smoking. The subjects were kept in the supine position in a quiet, dark, air-conditioned room (constant temperature of 22–26 °C) throughout the study. A 23-gauge polyethylene catheter was inserted into the left deep antecubital vein to obtain blood samples. ABI measurement and pulse wave recording were performed at least 5 min after maintaining the supine position. Vascular tests were performed by skilled and trained physicians without detailed knowledge of baseline clinical characteristics of the subjects.

### ABI measurement and pulse volume recording

ABI measurement and pulse volume recording for calculating %MAP were performed using a volume-plethysmographic apparatus (Form PWV/ABI, Omron Health Care Co., Kyoto, Japan). Four oscillometric cuffs were wrapped around both upper arms and lower legs. The cuffs were connected to an oscillometric pressure sensor for measurements of blood pressure and to a plethysmographic sensor for pulse volume recordings. Blood pressure in each limb was automatically and simultaneously measured, and then waveforms of pulse volume recording in the lower limbs were automatically and simultaneously recorded. This device distinguished between pulses of the anterior tibial artery, posterior tibial artery, and peroneal artery by using frequency analysis and automatically selected and displayed the pulse with the highest oscillation. ABI was automatically calculated by dividing the ankle systolic blood pressure of the right and left sides by the higher brachial systolic blood pressure of either arm.

**Fig. 1:**
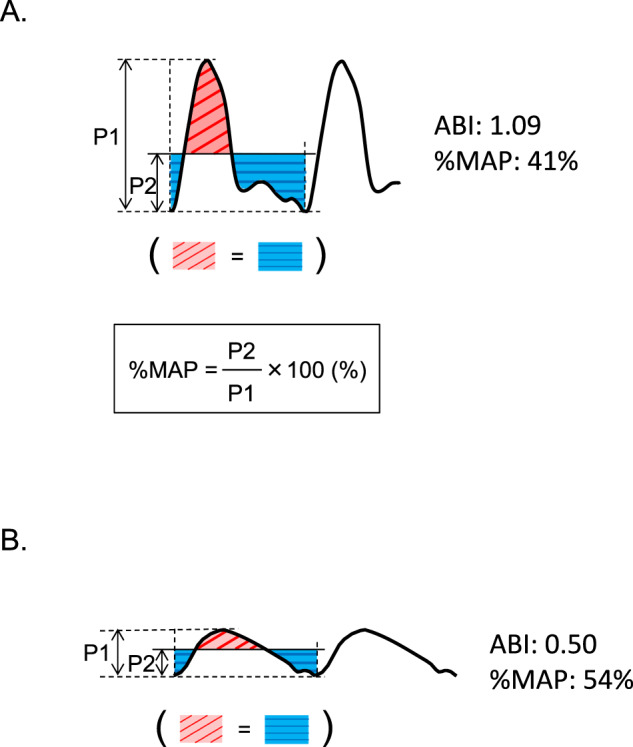
Waveforms of pulse volume recording and percentage of mean arterial pressure (%MAP). Waveforms of pulse volume recording with normal %MAP (**A**) and high %MAP (**B**)

Waveforms of pulse volume recording were obtained by holding cuff pressure at 54 mmHg in subjects with diastolic blood pressure above 62 mmHg and by holding cuff pressure at 8 mmHg below diastolic blood pressure in subjects with diastolic blood pressure below 62 mmHg to minimize the influence of cuff pressure on hemodynamics. Pulse waveforms in the lower limbs were recorded and stored for 10 s. The %MAP was automatically calculated for each pulse waveform and the mean of %MAPs obtained in the 10-second recording was used for analyses. A beat with a pulse interval 25% shorter or longer than the previous beat interval was excluded due to the possibility of arrhythmia or body movement. The %MAP was not calculated when the number of available pulses for calculation was less than three [10]. The %MAP is the height of the mean area of the arterial waveform (P2) divided by the peak amplitude (P1) [P2/P1 × 100 (%)] (Fig. 1A). The arterial waveform should be flattened and %MAP should increase with hemodynamically significant occlusive lesions in the lower extremity artery (Fig. 1B). The reproducibility of ABI and %MAP (on visit 1 and visit 2) was assessed in 30 consecutive subjects without medication change in whom ABI and %MAP were measured twice with a six-month interval by the same experienced observer. Pearson’s correlation coefficients of ABI between visit 1 and visit 2 were 0.59 (*p* < 0.001) in the right leg and 0.45 (*p* = 0.01) in the left leg, and the coefficients of variation were 4.0% in the right leg and 4.6% in the left leg. Pearson’s correlation coefficients of %MAP between visit 1 and 2 were 0.47 (*p* = 0.009) in the right leg and 0.40 (*p* = 0.03) in the left leg, and the coefficients of variation were 4.7% in the right leg and 5.7% in the left leg.

### Cutoff values of ABI and %MAP

The 2016 American Heart Association/American College of Cardiology guidelines on the management of peripheral artery disease (PAD) recommend that ABI in the range of 1.00–1.40 should be classified as normal for the diagnosis of LEAD [18]. In addition, a previous study showed that low ABI (<1.00) was associated with a higher incidence of cardiovascular events than was normal ABI (1.00–1.40) [6]. Therefore, participants were divided into two groups based on the cutoff value of ABI: subjects with low ABI when ABI on either side was <1.00 and subjects with normal ABI when ABIs on both the left and right sides were ≥1.00.

To our knowledge, there has been no report in which a cutoff value of %MAP for cardiovascular disease was proposed in the general population. Therefore, in accordance with the recommended cutoff value of %MAP for diagnosis of LEAD in the 2013 Japanese Circulation Society guidelines for a noninvasive vascular function test [11] and in the 2022 Japanese Circulation Society guideline on the management of PAD [12] and a cutoff value of %MAP proposed from the results of a recent study in which the optimal cutoff value of %MAP for diagnosis of LEAD was investigated [10], participants were divided into two groups: subjects with %MAP < 45% when the %MAPs on both the left and right sides were <45% and subjects with %MAP ≥ 45% when %MAP on either side or %MAPs on both sides were ≥45%. In the present study, these cutoff values of ABI and %MAP were used for severity assessment of atherosclerosis but not for diagnosis of LEAD.

### Statistical analysis

All reported *p* values were 2-sided, and a *p* value of <0.05 was considered statistically significant. Continuous variables are summarized as means ± standard deviation and were compared by using unpaired Student *t* test. Categorical variables are presented as frequencies and percentages and were compared by means of the chi-square test. The Cochran–Amitage trend test was used to assess the trend of ordered categorical variables for the association between ABI and proportion of subjects with high %MAP and the association between %MAP and prevalence of CAD. Multiple logistic regression analyses were performed to identify independent variables associated with high %MAP or CAD. Age, sex, body mass index (BMI), heart rate, hypertension, dyslipidemia, diabetes mellitus, smoking, hemodialysis, and ABI were entered as covariates into the model of the relationships between %MAP and variables. Age, sex, BMI, heart rate, hypertension, dyslipidemia, diabetes mellitus, smoking, and ABI were entered into the model for the association between CAD and %MAP. Multivariate analysis was performed among subjects with low ABI. Age and sex were entered into the model as covariates. To assess the diagnostic accuracy for clinical CAD, receiver operating characteristic (ROC) curve analyses were performed. The optimal cutoff value of %MAP was determined according to the highest Youden index from the ROC curve to diagnose clinical CAD. The differences in area under the curve (AUC) were compared using the method of Delong et al. [19]. The data were processed using JMP version pro 16 (SAS Institute, Cary, NC).

## Results

### Baseline clinical characteristics

**Fig. 2:**
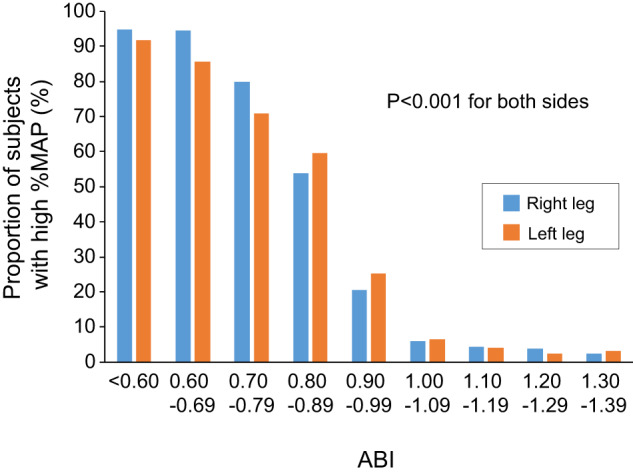
Bar graphs show the proportions of subjects with a percentage of mean arterial pressure (%MAP) ≥ 45% according to ankle-brachial index (ABI) in right and left legs

The baseline clinical characteristics of the subjects are summarized in Table 1. Of the 2213 participants, 304 (13.7%) had CAD. Mean values were 1.14 ± 0.12 for right ABI, 1.13 ± 0.13 for left ABI, 38.4 ± 4.7% for right %MAP, and 38.4 ± 4.5% for left %MAP. The proportion of patients with %MAP ≥ 45% was increased in relation to a decrease in ABI in both the left and right legs (*p* < 0.001) (Fig. 2). Multiple logistic regression analyses revealed that lower BMI, higher heart rate, diabetes mellitus, hemodialysis, and lower ABI were significantly associated with %MAP ≥ 45% in both the left and right legs (Supplementary Table 1).

**Table 1:** Clinical characteristics of subjects

	All	Low ABI	Normal ABI		Normal %MAP	High %MAP	
Variables	(*n* = 2213)	(*n* = 268)	(*n* = 1945)	*p* value	(*n* = 1972)	(*n* = 241)	*p* value
Age, y	61.2 ± 15.5	64.4 ± 16.3	60.8 ± 15.3	<0.001	60.8 ± 15.4	64.5 ± 15.5	<0.001
Male, *n* (%)	1361 (61.5)	171 (63.8)	1191 (61.2)	0.42	1222 (62.0)	140 (58.1)	0.24
Body mass index, kg/m^2^	23.9 ± 4.0	22.9 ± 4.2	24.1 ± 3.9	<0.001	24.1 ± 3.9	22.8 ± 4.0	<0.001
Systolic blood pressure, mmHg	132.7 ± 18.8	134.7 ± 21.0	132.4 ± 18.5	0.06	132.2 ± 18.3	136.6 ± 22.5	<0.001
Diastolic blood pressure, mmHg	78.4 ± 12.5	75.4 ± 12.2	78.8 ± 12.5	<0.001	78.6 ± 12.3	76.4 ± 13.4	0.01
Heart rate, bpm	69.5 ± 12.2	74.3 ± 13.3	68.9 ± 11.9	<0.001	69.0 ± 12.1	73.9 ± 12.4	<0.001
Total cholesterol, mmol/L	4.94 ± 0.96	4.75 ± 0.97	4.97 ± 0.96	0.002	4.96 ± 0.96	4.82 ± 1.02	0.05
Triglycerides, mmol/L	1.60 ± 1.21	1.57 ± 0.99	1.60 ± 1.24	0.71	1.60 ± 1.19	1.57 ± 1.36	0.68
HDL cholesterol, mmol/L	1.54 ± 0.44	1.54 ± 0.52	1.54 ± 0.43	0.93	1.53 ± 0.43	1.58 ± 0.54	0.15
LDL cholesterol, mmol/L	2.84 ± 0.84	2.62 ± 0.83	2.87 ± 0.84	<0.001	2.86 ± 0.84	2.66 ± 0.82	<0.001
Glucose, mmol/L	6.39 ± 2.23	7.61 ± 3.54	6.23 ± 1.94	<0.001	6.25 ± 1.98	7.62 ± 3.53	<0.001
HbA1c, %	5.9 ± 0.9	6.5 ± 1.3	5.9 ± 0.8	<0.001	5.9 ± 0.9	6.5 ± 1.4	<0.001
Creatinine, μmol/L	81.5 ± 73.5	112.3 ± 132.4	77.4 ± 60.6	<0.001	76.6 ± 55.1	123.9 ± 154.4	<0.001
Smoking, *n* (%)	1188 (54.0)	169 (63.5)	1019 (52.7)	<0.001	1049 (53.5)	139 (57.9)	0.20
Comorbidities, *n* (%)							
Hypertension	1824 (82.4)	213 (79.5)	1611 (82.8)	0.18	1633 (82.8)	191 (79.3)	0.17
Dyslipidemia	1596 (72.2)	208 (77.6)	1388 (71.4)	0.03	1422 (72.2)	174 (72.2)	0.99
Diabetes mellitus	624 (28.2)	119 (44.4)	505 (26.0)	<0.001	521 (26.4)	103 (42.7)	<0.001
Coronary artery disease	304 (13.7)	66 (24.6)	238 (12.2)	<0.001	250 (12.7)	54 (22.4)	<0.001
Previous myocardial infarction	123 (5.6)	36 (13.5)	87 (4.5)	<0.001	95 (4.8)	28 (11.7)	<0.001
Angina pectoris	252 (11.4)	49 (18.3)	203 (10.4)	<0.001	213 (10.8)	39 (16.2)	0.01
Prior coronary revascularization procedure	241 (10.9)	52 (19.4)	189 (9.7)	<0.001	199 (10.1)	42 (17.4)	<0.001
Hemodialysis	27 (1.2)	15 (5.6)	12 (0.6)	<0.001	12 (0.6)	15 (6.2)	<0.001
Medications, *n* (%)							
Antihypertensive drugs	1498 (67.7)	175 (65.3)	1323 (68.0)	0.37	1339 (67.9)	159 (66.0)	0.55
Lipid-lowering drugs	834 (37.7)	137 (51.1)	697 (35.8)	<0.001	727 (36.9)	107 (44.4)	0.02
Antidiabetic drugs	446 (20.2)	94 (35.1)	352 (18.1)	<0.001	359 (18.1)	87 (36.1)	<0.001
Right ABI	1.14 ± 0.12	0.93 ± 0.18	1.17 ± 0.08	NA	1.17 ± 0.11	0.99 ± 0.21	<0.001
Left ABI	1.13 ± 0.13	0.90 ± 0.16	1.17 ± 0.08	NA	1.15 ± 0.09	0.97 ± 0.21	<0.001
Right %MAP, %	38.4 ± 4.7	43.4 ± 6.8	37.8 ± 3.9	<0.001	37.4 ± 3.5	46.9 ± 4.8	NA
Left %MAP, %	38.4 ± 4.5	43.2 ± 6.1	37.8 ± 3.9	<0.001	37.4 ± 3.5	46.5 ± 3.9	NA

*ABI* indicates ankle-brachial index, *%MAP* percentage of mean arterial pressure, *HDL* high-density lipoprotein, *LDL* low-density lipoprotein, *HbA1c* hemoglobin A1c, *NA* not applicable

### Association between ABI and CAD

The lower ABI value of either side was used for analysis. The prevalence of CAD according to ABI had a reverse J-shaped distribution (Supplementary Fig. 1). We divided the participants into two groups according to ABI (low ABI: ABI < 1.00, normal ABI: ABI ≥ 1.00). The baseline clinical characteristics are summarized in Table 1. Of the 2213 subjects, 268 subjects (12.1%) had low ABI. The prevalence of CAD was significantly higher in subjects with low ABI than in subjects with normal ABI (24.6% vs. 12.2%, *p* < 0.001) (Table 1). Multiple logistic regression analysis revealed that low ABI was significantly associated with a higher risk of CAD (odds ratio [OR], 1.85; 95% confidence interval [CI], 1.27–2.69; *p* = 0.001) after adjustments for other traditional cardiovascular risk factors (Supplementary Table 2).

### Association between %MAP and CAD

**Fig. 3:**
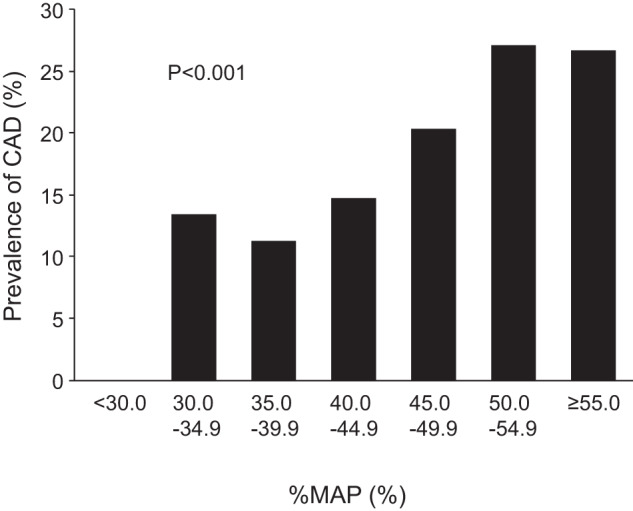
Bar graphs show the prevalence of coronary artery disease (CAD) according to percentage of mean arterial pressure (%MAP)

The higher %MAP value of either side was used for analysis. The prevalence of CAD increased in relation to an increase in %MAP (*p* < 0.001) (Fig. 3). We divided the participants into two groups according to %MAP: subjects with %MAP < 45% and patients with %MAP ≥ 45%. The baseline clinical characteristics are summarized in Table 1. Of the 2213 subjects, 241 subjects (10.9%) had %MAP ≥ 45%. The prevalence of CAD was significantly higher in patients with %MAP ≥ 45% than in subjects with %MAP < 45% (22.4% vs. 12.7%, *p* < 0.001) (Table 1). In an unadjusted analysis of the relationship between CAD and %MAP ≥ 45%, %MAP ≥ 45% was significantly associated with a higher risk of CAD (OR, 1.98; 95% CI, 1.43–2.77; *p* < 0.001) (Table 2, unadjusted model). In a multiple logistic regression analysis of relationships between CAD and variables, %MAP ≥ 45% was significantly associated with a higher risk of CAD after adjusting for traditional cardiovascular risk factors (OR, 2.14; 95% CI, 1.43–3.21; *p* < 0.001) (Table 2, Model 2). When the ABI value was entered into the model, there was no significant association between %MAP ≥ 45% and CAD (OR, 1.39; 95% CI, 0.83–2.33; *p* = 0.21), whereas ABI was significantly associated with CAD (OR, 0.98; 95% CI, 0.97–0.99; *p* = 0.005) (Table 2, Model 3). When systolic blood pressure at the time of %MAP measurement and antihypertensive drug treatment were entered instead of hypertension into the model of the relationships between %MAP ≥ 45% and variables, the insignificant association between %MAP ≥ 45% and CAD (OR, 1.30; 95% CI, 0.77–2.19, *p* = 0.33) and the significant association between ABI and CAD (OR, 0.98; 95% CI, 0.97–0.99, *p* = 0.001) remained unchanged (Table 2, Model 4). The AUC value of the ROC curve for ABI < 1.00 to diagnose clinical CAD was 0.56 (95% CI, 0.53–0.58) and that for %MAP ≥ 45% was 0.54 (95% CI, 0.52–0.56). The addition of %MAP ≥ 45% to ABI < 1.00 did not improve the diagnostic accuracy for clinical CAD [AUC: 0.56 (95% CI, 0.53–0.58) to 0.56 (95% CI, 0.53–0.58), *p* = 0.79].

**Table 2:** Association between coronary artery disease and %MAP ≥ 45% %MAP indicates percentage of mean arterial pressure; ABI, ankle-brachial index

	Odds ratio (95% Confidence Interval); *p* value				
Variable	Unadjusted	Model 1	Model 2	Model 3	Model 4
%MAP ≥ 45% (yes/no)	1.98 (1.43–2.77); <0.001	1.82 (1.28–2.61); 0.001	2.14 (1.43–3.21); <0.001	1.39 (0.83–2.33); 0.21	1.30 (0.77–2.19); 0.33
Age (year)	-	1.06 (1.05–1.07); <0.001	1.05 (1.04–1.07); <0.001	1.05 (1.03–1.06); <0.001	1.04 (1.03–1.06); <0.001
Male (yes/no)	-	3.99 (2.91–5.46); <0.001	3.48 (2.35–5.15); <0.001	3.50 (2.36–5.20); <0.001	3.33 (2.25–4.95); <0.001
Body mass index (kg/m^2^)	-	-	0.95 (0.91–0.99); 0.02	0.96 (0.92–0.99); 0.04	0.95 (0.92–0.99); 0.04
Heart rate (bpm)	-	-	0.96 (0.95–0.98); <0.001	0.96 (0.95–0.97); <0.001	0.96 (0.95–0.97); <0.001
Hypertension (yes/no)	-	-	1.53 (0.96–2.46); 0.07	1.58 (0.99–2.54); 0.06	-
Systolic blood pressure (mmHg)			-	-	0.99 (0.98–0.99); 0.04
Antihypertensive drug treatment (yes/no)			-	-	1.48 (1.03–2.11); 0.03
Dyslipidemia (yes/no)	-	-	12.3 (6.68–22.7); <0.001	12.7 (6.86–23.6); <0.001	12.0 (6.44–22.2); <0.001
Diabetes mellitus (yes/no)	-	-	2.40 (1.80–3.19); <0.001	2.33 (1.75–3.10); <0.001	2.37 (1.77–3.16); <0.001
Smoking (yes/no)	-	-	2.00 (1.41–2.86); <0.001	1.93 (1.35–2.76); <0.001	2.03 (1.42–2.91); <0.001
ABI (×100)	-	-	-	0.98 (0.97–0.99); 0.005	0.98 (0.97–0.99); 0.001

When the diagnostic accuracy of %MAP and that of ABI were compared as continuous values, there was no significant difference between the AUC value of %MAP to diagnose CAD and that of ABI [AUC: 0.56 (95% CI, 0.53–0.60) vs. 0.59 (95% CI, 0.55–0.63), *p* = 0.19]. The addition of %MAP to ABI as a continuous value did not improve the diagnostic accuracy for clinical CAD [AUC: 0.59 (95% CI, 0.55–0.63) to 0.59 (95% CI, 0.56–0.63), *p* = 0.51].

### Association between %MAP and CAD in subjects with normal ABI

We divided the subjects with normal ABI (≥1.00) into two groups according to the cutoff value of 45% for %MAP. The clinical characteristics are summarized in Supplementary Table 3. Of the 1945 subjects with normal ABI, 99 (5.1%) had %MAP ≥ 45%. There was no significant difference in the prevalence of CAD between subjects with %MAP < 45% and subjects with %MAP ≥ 45% (12.2% vs. 12.1%, *p* = 0.97) among the subjects with normal ABI (Supplementary Table 3).

### Association between %MAP and CAD in patients with low ABI

We divided the subjects with low ABI (<1.00) into two groups according to the cutoff value of 45% for %MAP. The clinical characteristics are summarized in Supplementary Table 4. Of the 268 patients with low ABI, 142 (53.0%) had %MAP ≥ 45%. The prevalence of CAD was significantly higher in patients with %MAP ≥ 45% than in patients with %MAP < 45% (29.6% vs. 19.1%, *p* = 0.04) (Supplementary Table 4). In an unadjusted analysis of the relationship between CAD and %MAP ≥ 45%, %MAP ≥ 45% was significantly associated with a higher risk of CAD (OR, 1.79; 95% CI, 1.01–3.16; *p* = 0.04) (Supplementary Table 5, unadjusted model). In a multiple logistic regression analysis of relationships between CAD and variables, %MAP ≥ 45% was not associated with CAD after adjusting for age and sex (OR, 1.60; 95% CI, 0.86–2.98; *p* = 0.14) (Supplementary Table 5, Model 1).

### Associations between ABI ≤ 0.90, %MAP, and CAD

To determine whether the cutoff value of the ABI affect the usefulness of %MAP, we divided the participants into two groups according to a cutoff value of 0.90 for ABI. The baseline clinical characteristics are summarized in Supplementary Table 6. Of the 2213 subjects, 142 subjects (6.4%) had ABI ≤ 0.90. The prevalence of CAD was significantly higher in subjects with ABI ≤ 0.90 than in subjects with ABI > 0.90 (31.7% vs. 12.5%, *p* < 0.001) (Supplementary Table 6). Multiple logistic regression analysis revealed that ABI ≤ 0.90 was significantly associated with a higher risk of CAD (OR, 2.89; 95% CI, 1.82–4.59; *p* < 0.001) after adjustments for other traditional cardiovascular risk factors (Supplementary Table 7).

We divided the subjects with ABI > 0.90 into two groups according to a cutoff value of 45% for %MAP. The clinical characteristics are summarized in Supplementary Table 8. Of the 2071 subjects with ABI > 0.90, 132 (6.4%) had %MAP ≥ 45%. There was no significant difference in the prevalence of CAD between subjects with %MAP < 45% and subjects with %MAP ≥ 45% (12.4% vs. 13.6%, *p* = 0.68). We divided the subjects with ABI ≤ 0.90 into two groups according to a cutoff value of 45% for %MAP. The clinical characteristics are summarized in Supplementary Table 9. Of the 142 subjects with ABI ≤ 0.90, 109 (76.8%) had %MAP ≥ 45%. There was no significant difference in the prevalence of CAD between subjects with %MAP < 45% and subjects with %MAP ≥ 45% (27.3% vs. 33.0%, *p* = 0.53).

### The optimal cutoff value of %MAP

The optimal cutoff value of %MAP derived from an ROC curve to diagnose clinical CAD was 40.3%. Subjects were divided into two groups according to the cutoff value of %MAP: subjects with %MAP < 40.3% (*n* = 1410) and subjects with %MAP ≥ 40.3% (*n* = 803). The clinical characteristics of subjects according to the cutoff value of %MAP are summarized in Supplementary Table 10. The prevalence of CAD was significantly higher in subjects with %MAP ≥ 40.3% than in subjects with <40.3% (18.2% vs. 11.2%, *p* < 0.001). In an unadjusted analysis of the relationship between CAD and %MAP ≥ 40.3%, %MAP ≥ 40.3% was significantly associated with a higher risk of CAD (OR, 1.76; 95% CI, 1.38–2.25; *p* < 0.001) (Supplementary Table 11). When the ABI value was entered into the model, the association between %MAP ≥ 40.3% and CAD remained significant (OR, 1.63; 95% CI, 1.19–2.24; *p* = 0.002) (Supplementary Table 11, Model 3). The AUC value of the ROC curve for %MAP ≥ 40.3% was 0.57 (95% CI, 0.54–0.60). The addition of %MAP ≥ 40.4% to ABI < 1.00 significantly improved the diagnostic accuracy for clinical CAD [AUC: 0.56 (95% CI, 0.53–0.58) to 0.59 (95% CI, 0.56–0.62), *p* = 0.006].

## Discussion

In the present study, we showed that lower ABI was significantly associated with a higher risk of clinical CAD. In addition, the proportion of patients with clinical CAD was significantly higher in patients with %MAP ≥ 45% than in patients with %MAP < 45%. Although we found a significant association between %MAP ≥ 45% and CAD in an unadjusted analysis, the association was attenuated and no longer significant after adjusting for traditional cardiovascular risk factors and ABI. Moreover, subgroup analyses showed that %MAP ≥ 45% was not associated with CAD in either subjects with normal ABI or subjects with low ABI. Furthermore, when a cutoff value of 0.9 for ABI was used, %MAP ≥ 45% was not associated with CAD in either subjects with ABI > 0.90 or subjects with ABI ≤ 0.90. However, %MAP ≥ 40.3%, the optimal cutoff value derived from an ROC curve to diagnose clinical CAD, was significantly associated with CAD even after adjusting traditional risk factors and ABI. These findings suggest that the cutoff value of 40.3%, but not 45% recommended for LEAD screening, is useful for cardiovascular risk assessment even when the ABI value is taken into account.

ABI has been used not only for the diagnosis and severity assessment of LEAD but also for cardiovascular risk assessment since ABI is not only an indicator of occlusive arterial lesions in the lower extremities but also an indicator of generalized atherosclerosis and cardiovascular prognosis. The results of previous studies showed that lower ABI is associated with a higher risk of clinical cardiovascular disease and higher incidence of cardiovascular events and that cardiovascular risk increases with decreasing ABI [4–6, 20, 21]. Indeed, the results of the present study showed that the proportion of patients with clinical CAD increased with decreasing ABI and that low ABI (<1.00) was significantly associated with CAD independent of traditional cardiovascular risk factors, suggesting that ABI is a useful vascular marker for cardiovascular risk assessment. However, ABI is not always reliable since ABI can be falsely normalized despite the presence of occlusive arterial lesions in the lower extremities in patients with calcified noncompressible lower limb arteries due to falsely elevated ankle systolic blood pressure, which can lead to underestimation of cardiovascular risk. Therefore, other vascular markers should be combined with ABI to improve diagnostic accuracy of ABI for cardiovascular risk assessment [22, 23].

Volume change in the lower limb generated by pulsatile artery inflow can be recorded by using plethysmography. Technological advances in pnuemoplethysmography using the cuff method have made it possible to obtain accurate pulse volume waveforms at the ankle in a short time during ABI measurement, and pulse volume recording parameters, including %MAP, are automatically calculated by an automated oscillometric device, which can lead to objective evaluation and clinical application of pulse volume recording parameters. The %MAP is the height of the mean area of the arterial waveform divided by the peak amplitude. In patients with hemodynamically occlusive lesions in the lower extremity artery, the pulse volume waveforms at the ankle tend to be blunted and, consequently, %MAP should be increased. Indeed, the proportion of patients with high %MAP increased with decreasing ABI in the present study. Although the results of a previous study showed that a combination of %MAP and ABI improves diagnostic accuracy for LEAD compared with ABI alone [10], there is little information on whether %MAP alone or in combination with ABI is useful for cardiovascular risk assessment as a marker of atherosclerosis. In the present study, the proportion of patients with clinical CAD was significantly higher in patients with %MAP ≥ 45% than in patients with %MAP < 45%. However, multivariate analysis revealed that there was no significant association between %MAP ≥ 45% and CAD after adjusting for several confounding factors, including ABI, whereas ABI was significantly associated with clinical CAD. These findings suggest that the cutoff value of 45% for %MAP recommended for the diagnosis of LEAD is not useful for cardiovascular risk assessment.

The optimal cutoff value of %MAP derived from the ROC curve to diagnose patients with clinical CAD was 40.3%. The proportion of patients with clinical CAD was significantly higher in patients with %MAP ≥ 40.3% than in patients with %MAP < 40.3% and %MAP ≥ 40.3% was significantly associated with CAD independent of ABI. Moreover, the addition of %MAP ≥ 40.3% to ABI < 1.00 improved the diagnostic accuracy for clinical CAD. These findings suggest that the cutoff value of 40.3%, but not 45% recommended for LEAD screening, for %MAP is useful for identifying patients with advanced atherosclerosis and cardiovascular risk assessment independent of ABI and that paying attention to whether %MAP is greater than 40.3% or not may reduce the risk of missing patients with advanced atherosclerosis. The optimal cutoff value of %MAP for clinical CAD may be lower than that for LEAD screening, which may be related to the insignificant association between %MAP ≥ 45% and clinical CAD.

The usefulness of %MAP as a prognostic marker has been investigated in some clinical studies [24–26]. Li et al. showed that high %MAP ( > 45%) was significantly associated with a higher risk of all-cause mortality in subjects with normal ABI (0.9 < ABI ≤ 1.3) during a mean follow-up period of 20.3 months in an observational study in which almost 80% of the participants had diabetes mellitus [24]. The same investigators also reported that patients with a combination of high %MAP (>45%) and normal ABI (>0.9) had a significantly higher risk of all-cause mortality than did patients with a combination of normal %MAP (≤45%) and normal ABI (>0.9) among patients with type 2 diabetes during a median follow-up period of 22.9 months [26]. Lee et al. reported that %MAP > 50% was significantly associated with higher all-cause mortality and cardiovascular mortality in patients who were receiving chronic hemodialysis during a mean follow-up period of 2.7 years [25]. These findings suggest that %MAP is useful for the assessment of mortality risk and cardiovascular risk in patients with type 2 diabetes mellitus and/or end-stage renal disease. The results of the present study support the findings of the previous studies, although the cutoff values of %MAP were different among the studies.

The device used for the measurements of ABI and %MAP in this study is now widely adopted in Asia, especially in parts of East Asia. Measurements with this device are operator-independent, noninvasive, and can be performed in a relatively short time. Considering the usefulness of ABI and %MAP for cardiovascular risk assessment, the measurement of ABI and %MAP should be performed more aggressively not only for LEAD screening but also for cardiovascular risk assessment in patients with cardiovascular risk factors in daily clinical practice.

There are several limitations in the present study. First, the possibility of the presence of residual unmeasured confounding factors cannot be excluded. Second, the results cannot be generalized to individuals with an ABI ≥ 1.4 since subjects with an ABI ≥ 1.4 were excluded from this study. Third, it remains unclear whether %MAP is a useful vascular marker for predicting future cardiovascular events because this study was a cross-sectional study. Fourth, although diagnosis of CAD was confirmed by CAG in all patients with clinical CAD, not all subjects without clinical CAD underwent CAG. Information on whether CAG was performed was not available and the exact number of subjects who underwent CAG among subjects without clinical CAD is unclear. Therefore, we cannot deny the possibility that patients without clinical CAD had latent coronary artery stenosis. Fifth, information on the exact date of CAG was not available. Therefore, the exact time interval between CAG and %MAP measurement was unclear. We cannot deny the possibility that %MAP did not reflect the condition of the lower extremity arteries at the time of diagnosis of CAD due to the time interval between CAG and %MAP measurement and that the time interval affected the association between %MAP and clinical CAD.

In conclusion, the cutoff value of 40.3%, but not 45% recommended for the diagnosis of LEAD, for %MAP may be useful for detecting patients with advanced atherosclerosis even when the ABI value is taken into account for cardiovascular risk assessment.

### Supplementary information

Supplementary Information
