# Supplementary material for: Percentage of mean arterial pressure as a marker of atherosclerosis for detecting patients with coronary artery disease
Source: Hypertens Res. 2023 Oct 4;47(2):281–90. doi: 10.1038/s41440-023-01442-4 (PMC10838767; doi:10.1038/s41440-023-01442-4)
Supplement: Supplementary file 1 — Supplementary Information [file 41440_2023_1442_MOESM1_ESM.docx]

ONLINE SUPPLEMENT

**Percentage of Mean Arterial Pressure As A Marker of Atherosclerosis for Detecting Patients with Coronary Artery Disease**

Brief title: ％MAP and coronary artery disease

Tatsuya Maruhashi, MD, PhD;^1^ Masato Kajikawa, MD, PhD;^2^ Shinji Kishimoto, MD, PhD;^1^ Takayuki Yamaji, MD, PhD;^1^ Takahiro Harada, MD, PhD;^1^ Yu Hashimoto, MD;^3^ Aya Mizobuchi, MS;^1^ Shunsuke Tanigawa, MS;^1^ Farina Mohamad Yusoff, MD, PhD;^1^ Yukiko Nakano, MD, PhD;^3^ Kazuaki Chayama, MD, PhD;^4^ Ayumu Nakashima, MD, PhD;^5^ Chikara Goto, PhD;^6^ Yukihito Higashi, MD, PhD^1, 2^

^1^Department of Regenerative Medicine, Research Institute for Radiation Biology and Medicine, Hiroshima University, 1-2-3 Kasumi, Minami-ku, Hiroshima, Japan

^2^Division of Regeneration and Medicine, Medical Center for Translational and Clinical Research, Hiroshima University Hospital, 1-2-3 Kasumi, Minami-ku, Hiroshima, Japan

^3^Department of Cardiovascular Medicine, Graduate School of Biomedical and Health Sciences, Hiroshima University, 1-2-3 Kasumi, Minami-ku, Hiroshima, Japan

^4^Department of Medicine and Molecular Science, Hiroshima University Graduate School of Biomedical Sciences, Hiroshima University, 1-2-3 Kasumi, Minami-ku, Hiroshima, Japan

^5^Department of Stem Cell Biology and Medicine, Graduate School of Biomedical and Sciences, Hiroshima University, 1-2-3 Kasumi, Minami-ku, Hiroshima, Japan

^6^Department of Rehabilitation, Faculty of general Rehabilitation, Hiroshima International University, 555-36, Kurosegakuendai, Higashihiroshima, Japan

Address for correspondence: Yukihito Higashi, MD, PhD, FAHA

Department of Regenerative Medicine

Research Institute for Radiation Biology and Medicine, Hiroshima University

1-2-3 Kasumi, Minami-ku, Hiroshima 734-8551, Japan

Phone: +81-82-257-5831 Fax: +81-82-257-5831

E-mail: [yhigashi@hiroshima-u.ac.jp](mailto:yhigashi@hiroshima-u.ac.jp)

**Supplementary Table 1.** Multiple Logistic Regression Analysis of the Relationships Between %MAP ≥45% and Variables in the Right Ankle and Left Ankle

| Covariates | Right ankle | |  | Left ankle | |
| --- | --- | --- | --- | --- | --- |
|  | Odds ratio (95% CI) | *p* value |  | Odds ratio (95% CI) | *p* value |
| Age (year) | 1.01 (0.99-1.02) | 0.27 |  | 1.00 (0.99-1.02) | 0.65 |
| Male (yes/no) | 0.74 (0.47-1.20) | 0.23 |  | 0.70 (0.44-1.11) | 0.13 |
| Body mass index (kg/m^2^) | 0.95 (0.90-0.99) | 0.04 |  | 0.93 (0.88-0.98) | 0.006 |
| Heart rate (bpm) | 1.01 (1.00-1.03) | 0.02 |  | 1.02 (1.01-1.04) | 0.001 |
| Hypertension (yes/no) | 0.78 (0.47-1.30) | 0.34 |  | 1.01 (0.61-1.69) | 0.97 |
| Dyslipidemia (yes/no) | 0.81 (0.52-1.27) | 0.36 |  | 0.93 (0.60-1.45) | 0.76 |
| Diabetes mellitus (yes/no) | 2.11 (1.41-3.16) | <0.001 |  | 1.56 (1.05-2.33) | 0.03 |
| Smoking (yes/no) | 1.25 (0.79-1.99) | 0.34 |  | 1.19 (0.75-1.89) | 0.46 |
| Hemodialysis (yes/no) | 6.70 (2.18-20.6) | <0.001 |  | 3.77 (1.31-10.8) | 0.01 |
| ABI (×100) | 0.92 (0.91-0.93) | <0.001 |  | 0.91 (0.90-0.92) | <0.001 |

ABI indicates ankle-brachial index; %MAP, percentage of mean arterial pressure.

**Supplementary Table 2.** Association between Coronary Artery Disease and Low ABI (<1.0)

|  | Odds ratio (95% Confidence Interval);  *p* value | | |
| --- | --- | --- | --- |
| Variable | Unadjusted | Model 1 | Model 2 |
| Low ABI (yes/no) | 2.34 (1.72-3.19);  <0.001 | 2.00 (1.43-2.79);  <0.001 | 1.85 (1.27-2.69);  0.001 |
| Age (year) | - | 1.06 (1.05-1.07);  <0.001 | 1.05 (1.04-1.07);  <0.001 |
| Male (yes/no) | - | 3.92 (2.86-5.37);  <0.001 | 3.38 (2.28-5.01);  <0.001 |
| Body mass index (kg/m^2^) | - | - | 0.95 (0.91-0.99);  0.02 |
| Heart rate (bpm) | - | - | 0.96 (0.95-0.98);  <0.001 |
| Hypertension (yes/no) | - | - | 1.50 (0.94-2.39);  0.09 |
| Dyslipidemia (yes/no) | - | - | 11.8 (6.40-21.6);  <0.001 |
| Diabetes mellitus (yes/no) | - | - | 2.35 (1.77-3.13);  <0.001 |
| Smoking (yes/no) | - | - | 1.96 (1.37-2.81);  <0.001 |

ABI indicates ankle-brachial index.

**Supplementary Table 3.** Clinical Characteristics of Subjects with Normal ABI According to %MAP of 45%

|  | %MAP  <45% | %MAP  ≥45% |  |
| --- | --- | --- | --- |
| Variables | (n=1846) | (n=99) | *p* value |
| Age, y | 60.9±15.3 | 58.5±15.8 | 0.12 |
| Male, n (%) | 1142 (61.9) | 49 (49.5) | 0.01 |
| Body mass index, kg/m^2^ | 24.1±3.9 | 23.3±4.0 | 0.06 |
| Systolic blood pressure, mm Hg | 132.2±18.1 | 137.4±24.1 | 0.007 |
| Diastolic blood pressure, mm Hg | 78.7±12.3 | 79.5±15.2 | 0.53 |
| Heart rate, bpm | 68.7±11.9 | 72.3±11.1 | 0.004 |
| Total cholesterol, mmol/L | 4.97±0.96 | 5.00±1.08 | 0.78 |
| Triglycerides, mmol/L | 1.60±1.21 | 1.59±1.75 | 0.92 |
| HDL cholesterol, mmol/L | 1.53±0.43 | 1.64±0.54 | 0.02 |
| LDL cholesterol, mmol/L | 2.88±0.83 | 2.80±0.85 | 0.42 |
| Glucose, mmol/L | 6.21±1.90 | 6.68±2.51 | 0.03 |
| HbA1c, % | 5.9±0.8 | 6.2±1.2 | 0.01 |
| Creatinine, μmol/L | 76.3±55.5 | 98.3±120.3 | <0.001 |
| Smoking, n (%) | 972 (53.0) | 47 (47.5) | 0.29 |
| Comorbidities, n (%) |  |  |  |
| Hypertension | 1534 (83.1) | 77 (77.8) | 0.17 |
| Dyslipidemia | 1323 (71.7) | 65 (65.7) | 0.19 |
| Diabetes mellitus | 474 (25.7) | 31 (31.3) | 0.21 |
| Coronary artery disease | 226 (12.2) | 12 (12.1) | 0.97 |
| Previous myocardial infarction | 83 (4.5) | 4 (4.0) | 0.83 |
| Angina pectoris | 191 (10.4) | 12 (12.1) | 0.57 |
| Prior coronary revascularization procedure | 180 (9.8) | 9 (9.1) | 0.83 |
| Hemodialysis | 8 (0.4) | 4 (4.0) | <0.001 |
| Medications, n (%) |  |  |  |
| Antihypertensive drugs | 1259 (68.2) | 64 (64.7) | 0.46 |
| Lipid-lowering drugs | 666 (36.1) | 31 (31.3) | 0.34 |
| Antidiabetic drugs | 327 (17.7) | 25 (25.3) | 0.06 |

ABI indicates ankle-brachial index; %MAP, percentage of mean arterial pressure; HDL, high-density lipoprotein; LDL, low-density lipoprotein; HbA1c, hemoglobin A1c.

**Supplementary Table 4.** Clinical Characteristics of Subjects with Low ABI According to %MAP of 45%

|  | %MAP  <45% | %MAP  ≥45% |  |
| --- | --- | --- | --- |
| Variables | (n=126) | (n=142) | *p* value |
| Age, y | 59.7±17.4 | 68.7±13.9 | <0.001 |
| Male, n (%) | 80 (63.5) | 91 (64.1) | 0.92 |
| Body mass index, kg/m^2^ | 23.5±4.4 | 22.4±3.9 | 0.04 |
| Systolic blood pressure, mm Hg | 133.2±20.4 | 136.0±21.5 | 0.27 |
| Diastolic blood pressure, mm Hg | 76.8±12.7 | 74.3±11.6 | 0.09 |
| Heart rate, bpm | 73.5±13.4 | 75.0±13.2 | 0.38 |
| Total cholesterol, mmol/L | 4.82±0.98 | 4.69±0.96 | 0.34 |
| Triglycerides, mmol/L | 1.59±0.99 | 1.55±0.99 | 0.73 |
| HDL cholesterol, mmol/L | 1.55±0.50 | 1.53±0.53 | 0.80 |
| LDL cholesterol, mmol/L | 2.69±0.88 | 2.56±0.78 | 0.24 |
| Glucose, mmol/L | 6.91±2.86 | 8.28±3.98 | 0.003 |
| HbA1c, % | 6.2±1.0 | 6.9±1.5 | 0.01 |
| Creatinine, μmol/L | 79.9±47.8 | 142.8±173.5 | <0.001 |
| Smoking, n (%) | 77 (61.6) | 92 (65.3) | 0.54 |
| Comorbidities, n (%) |  |  |  |
| Hypertension | 99 (78.6) | 114 (80.3) | 0.73 |
| Dyslipidemia | 99 (78.6) | 109 (76.8) | 0.72 |
| Diabetes mellitus | 47 (37.3) | 72 (50.7) | 0.03 |
| Coronary artery disease | 24 (19.1) | 42 (29.6) | 0.04 |
| Previous myocardial infarction | 12 (9.5) | 24 (17.0) | 0.07 |
| Angina pectoris | 22 (17.5) | 27 (19.0) | 0.74 |
| Prior coronary revascularization procedure | 19 (15.1) | 33 (23.2) | 0.09 |
| Hemodialysis | 4 (3.2) | 11 (7.8) | 0.11 |
| Medications, n (%) |  |  |  |
| Antihypertensive drugs | 80 (63.5) | 95 (66.9) | 0.56 |
| Lipid-lowering drugs | 61 (48.4) | 76 (53.5) | 0.40 |
| Antidiabetic drugs | 32 (25.4) | 62 (43.7) | 0.002 |

ABI indicates ankle-brachial index; %MAP, percentage of mean arterial pressure; HDL, high-density lipoprotein; LDL, low-density lipoprotein; HbA1c, hemoglobin A1c.

**Supplementary Table 5.** Association between Coronary Artery Disease and High %MAP (≥45%) in Patients with Low ABI

|  | Odds ratio (95% Confidence Interval);  *p* value | |
| --- | --- | --- |
| Variable | Unadjusted | Model 1 |
| High %MAP (yes/no) | 1.79 (1.01-3.16);  0.04 | 1.60 (0.86-2.98);  0.14 |
| Age (year) | - | 1.02 (0.99-1.04);  0.05 |
| Male (yes/no) | - | 7.42 (3.20-17.2);  <0.001 |

%MAP indicates percentage of mean arterial pressure; ABI, ankle-brachial index.

**Supplementary Table 6.** Clinical Characteristics of Subjects According to a Cutoff Value of 0.90 for ABI

|  | ABI >0.9 | ABI ≤0.9 |  |
| --- | --- | --- | --- |
| Variables | (n=2071) | (n=142) | *p* value |
| Age, y | 60.9±15.4 | 66.6±15.7 | <0.001 |
| Male, n (%) | 1262 (60.9) | 100 (70.4) | 0.02 |
| Body mass index, kg/m^2^ | 24.0±3.9 | 22.2±3.8 | <0.001 |
| Systolic blood pressure, mm Hg | 132.6±18.6 | 134.2±21.7 | 0.31 |
| Diastolic blood pressure, mm Hg | 78.7±12.5 | 73.6±11.6 | <0.001 |
| Heart rate, bpm | 69.2±12.0 | 75.0±13.5 | <0.001 |
| Total cholesterol, mmol/L | 4.96±0.96 | 4.69±0.99 | 0.003 |
| Triglycerides, mmol/L | 1.61±1.23 | 1.46±0.85 | 0.19 |
| HDL cholesterol, mmol/L | 1.54±0.43 | 1.55±0.56 | 0.75 |
| LDL cholesterol, mmol/L | 2.86±0.84 | 2.56±0.84 | <0.001 |
| Glucose, mmol/L | 6.27±1.99 | 8.32±4.04 | <0.001 |
| HbA1c, % | 5.9±0.9 | 6.8±1.6 | <0.001 |
| Creatinine, μmol/L | 77.4±59.5 | 144.1±173.5 | <0.001 |
| Smoking, n (%) | 1096 (53.1) | 95 (66.9) | 0.001 |
| Comorbidities, n (%) |  |  |  |
| Hypertension | 1713 (82.7) | 111 (78.2) | 0.17 |
| Dyslipidemia | 1491 (72.0) | 105 (73.9) | 0.62 |
| Diabetes mellitus | 559 (27.0) | 65 (45.8) | <0.001 |
| Coronary artery disease | 259 (12.5) | 45 (31.7) | <0.001 |
| Previous myocardial infarction | 99 (4.8) | 24 (17.0) | <0.001 |
| Angina pectoris | 221 (10.7) | 31 (21.8) | <0.001 |
| Prior coronary revascularization procedure | 207 (10.0) | 34 (23.9) | <0.001 |
| Hemodialysis | 13 (0.63) | 14 (9.9) | <0.001 |
| Medications, n (%) |  |  |  |
| Antihypertensive drugs | 1406 (67.9) | 92 (64.8) | 0.44 |
| Lipid-lowering drugs | 761 (36.8) | 73 (51.4) | <0.001 |
| Antidiabetic drugs | 390 (18.8) | 56 (39.4) | <0.001 |

ABI indicates ankle-brachial index; %MAP, percentage of mean arterial pressure; HDL, high-density lipoprotein; LDL, low-density lipoprotein; HbA1c, hemoglobin A1c.

**Supplementary Table 7.** Association between Coronary Artery Disease and ABI ≤0.90

|  | Odds ratio (95% Confidence Interval);  *p* value | | |
| --- | --- | --- | --- |
| Variable | Unadjusted | Model 1 | Model 2 |
| ABI ≤0.9 (yes/no) | 3.25 (2.23-4.73); <0.001 | 2.39 (1.59-3.59); <0.001 | 2.89 (1.82-4.59); <0.001 |
| Age (year) | - | 1.06 (1.05-1.07); <0.001 | 1.05 (1.04-1.06); <0.001 |
| Male (yes/no) | - | 3.83 (2.79-5.24); <0.001 | 3.29 (2.22-4.87); <0.001 |
| Body mass index (kg/m^2^) | - | - | 0.95 (0.91-0.99); 0.03 |
| Heart rate (bpm) | - | - | 0.96 (0.95-0.98); <0.001 |
| Hypertension (yes/no) | - | - | 1.56 (0.97-2.51); 0.06 |
| Dyslipidemia (yes/no) | - | - | 12.5 (6.76-23.0); <0.001 |
| Diabetes mellitus (yes/no) | - | - | 2.40 (1.81-3.19); <0.001 |
| Smoking (yes/no) | - | - | 2.00 (1.40-2.85); <0.001 |

ABI indicates ankle-brachial index.

**Supplementary Table 8.** Clinical Characteristics of Subjects with ABI >0.90 According to %MAP of 45%

|  | %MAP  <45% | %MAP  ≥45% |  |
| --- | --- | --- | --- |
| Variables | (n=1939) | (n=132) | *p* value |
| Age, y | 60.8±15.4 | 61.2±15.5 | 0.78 |
| Male, n (%) | 1196 (61.7) | 66 (50.0) | 0.008 |
| Body mass index, kg/m^2^ | 24.1±3.9 | 23.2±4.1 | 0.02 |
| Systolic blood pressure, mm Hg | 132.2±18.2 | 137.6±22.9 | 0.001 |
| Diastolic blood pressure, mm Hg | 78.7±12.3 | 78.9±14.2 | 0.86 |
| Heart rate, bpm | 68.9±12.0 | 73.2±11.3 | <0.001 |
| Total cholesterol, mmol/L | 4.96±0.96 | 4.95±1.04 | 0.93 |
| Triglycerides, mmol/L | 1.60±1.20 | 1.65±1.65 | 0.71 |
| HDL cholesterol, mmol/L | 1.53±0.43 | 1.63±0.53 | 0.02 |
| LDL cholesterol, mmol/L | 2.87±0.84 | 2.75±0.82 | 0.14 |
| Glucose, mmol/L | 6.22±1.92 | 6.95±2.83 | <0.001 |
| HbA1c, % | 5.9±0.8 | 6.3±1.1 | 0.002 |
| Creatinine, μmol/L | 76.2±54.4 | 96.6±110.1 | <0.001 |
| Smoking, n (%) | 1025 (53.2) | 68 (51.9) | 0.78 |
| Comorbidities, n (%) |  |  |  |
| Hypertension | 1608 (82.9) | 105 (79.6) | 0.32 |
| Dyslipidemia | 1398 (72.1) | 93 (70.5) | 0.68 |
| Diabetes mellitus | 510 (26.3) | 49 (37.1) | 0.007 |
| Coronary artery disease | 241 (12.4) | 18 (13.6) | 0.68 |
| Previous myocardial infarction | 92 (4.8) | 7 (5.3) | 0.77 |
| Angina pectoris | 206 (10.6) | 15 (11.4) | 0.79 |
| Prior coronary revascularization procedure | 193 (10.0) | 14 (10.6) | 0.81 |
| Hemodialysis | 9 (0.5) | 4 (3.0) | <0.001 |
| Medications, n (%) |  |  |  |
| Antihypertensive drugs | 1318 (68.0) | 88 (66.7) | 0.76 |
| Lipid-lowering drugs | 710 (36.6) | 51 (38.6) | 0.64 |
| Antidiabetic drugs | 350 (18.1) | 40 (30.3) | <0.001 |

ABI indicates ankle-brachial index; %MAP, percentage of mean arterial pressure; HDL, high-density lipoprotein; LDL, low-density lipoprotein; HbA1c, hemoglobin A1c.

**Supplementary Table 9.** Clinical Characteristics of Subjects with ABI ≤0.90 According to %MAP of 45%

|  | %MAP  <45% | %MAP  ≥45% |  |
| --- | --- | --- | --- |
| Variables | (n=33) | (n=109) | *p* value |
| Age, y | 60.6±17.5 | 68.5±14.7 | 0.01 |
| Male, n (%) | 26 (78.8) | 74 (67.9) | 0.23 |
| Body mass index, kg/m^2^ | 21.9±3.9 | 22.3±3.8 | 0.56 |
| Systolic blood pressure, mm Hg | 130.6±20.4 | 135.3±22.1 | 0.28 |
| Diastolic blood pressure, mm Hg | 74.0±11.3 | 73.4±11.7 | 0.82 |
| Heart rate, bpm | 76.2±13.0 | 74.7±13.7 | 0.58 |
| Total cholesterol, mmol/L | 4.80±1.05 | 4.66±0.98 | 0.53 |
| Triglycerides, mmol/L | 1.45±0.85 | 1.46±0.86 | 0.96 |
| HDL cholesterol, mmol/L | 1.66±0.60 | 1.52±0.55 | 0.23 |
| LDL cholesterol, mmol/L | 2.57±0.95 | 2.55±0.81 | 0.93 |
| Glucose, mmol/L | 8.01±3.90 | 8.43±4.10 | 0.63 |
| HbA1c, % | 6.5±1.5 | 7.0±1.7 | 0.33 |
| Creatinine, μmol/L | 100.2±84.0 | 158.0±191.6 | 0.11 |
| Smoking, n (%) | 24 (72.7) | 71 (65.1) | 0.42 |
| Comorbidities, n (%) |  |  |  |
| Hypertension | 25 (75.8) | 86 (78.9) | 0.70 |
| Dyslipidemia | 24 (72.7) | 81 (74.3) | 0.86 |
| Diabetes mellitus | 11 (33.3) | 54 (49.5) | 0.10 |
| Coronary artery disease | 9 (27.3) | 36 (33.0) | 0.53 |
| Previous myocardial infarction | 3 (9.1) | 21 (19.4) | 0.17 |
| Angina pectoris | 7 (21.2) | 24 (22.0) | 0.92 |
| Prior coronary revascularization procedure | 6 (18.2) | 28 (25.7) | 0.38 |
| Hemodialysis | 3 (9.1) | 11 (10.1) | 0.87 |
| Medications, n (%) |  |  |  |
| Antihypertensive drugs | 21 (63.6) | 71 (65.1) | 0.87 |
| Lipid-lowering drugs | 17 (51.5) | 56 (51.4) | 0.99 |
| Antidiabetic drugs | 9 (27.3) | 47 (43.1) | 0.10 |

ABI indicates ankle-brachial index; %MAP, percentage of mean arterial pressure; HDL, high-density lipoprotein; LDL, low-density lipoprotein; HbA1c, hemoglobin A1c.

**Supplementary Table 10.** Clinical Characteristics According to %MAP of 40.3%

|  | %MAP  <40.3% | %MAP  ≥40.3% |  |
| --- | --- | --- | --- |
| Variables | (n=1410) | (n=803) | *p* value |
| Age, y | 60.0±15.3 | 63.5±15.4 | <0.001 |
| Male, n (%) | 943 (66.9) | 419 (52.2) | <0.001 |
| Body mass index, kg/m^2^ | 24.2±3.9 | 23.4±4.0 | <0.001 |
| Systolic blood pressure, mm Hg | 131.1±18.0 | 135.5±19.8 | <0.001 |
| Diastolic blood pressure, mm Hg | 78.9±12.4 | 77.4±12.5 | 0.008 |
| Heart rate, bpm | 68.7±12.4 | 71.0±11.7 | <0.001 |
| Total cholesterol, mmol/L | 4.99±0.98 | 4.85±0.94 | 0.003 |
| Triglycerides, mmol/L | 1.64±1.27 | 1.53±1.09 | 0.04 |
| HDL cholesterol, mmol/L | 1.52±0.42 | 1.58±0.48 | 0.003 |
| LDL cholesterol, mmol/L | 2.90±0.84 | 2.74±0.82 | <0.001 |
| Glucose, mmol/L | 6.17±1.88 | 6.82±2.73 | <0.001 |
| HbA1c, % | 5.8±0.9 | 6.1±1.0 | <0.001 |
| Creatinine, μmol/L | 77.4±55.5 | 89.0±98.0 | <0.001 |
| Smoking, n (%) | 760 (54.3) | 428 (53.5) | 0.72 |
| Comorbidities, n (%) |  |  |  |
| Hypertension | 1169 (82.9) | 655 (81.6) | 0.43 |
| Dyslipidemia | 1013 (71.8) | 583 (72.7) | 0.67 |
| Diabetes mellitus | 346 (24.5) | 278 (34.7) | <0.001 |
| Coronary artery disease | 158 (11.2) | 146 (18.2) | <0.001 |
| Previous myocardial infarction | 64 (4.5) | 59 (7.4) | 0.005 |
| Angina pectoris | 135 (9.6) | 117 (14.6) | <0.001 |
| Prior coronary revascularization procedure | 127 (9.0) | 114 (14.2) | <0.001 |
| Hemodialysis | 6 (0.4) | 21 (2.6) | <0.001 |
| Medications, n (%) |  |  |  |
| Antihypertensive drugs | 958 (67.9) | 540 (67.3) | 0.74 |
| Lipid-lowering drugs | 483 (34.3) | 351 (43.7) | <0.001 |
| Antidiabetic drugs | 230 (16.3) | 216 (26.9) | <0.001 |

%MAP indicates percentage of mean arterial pressure; HDL, high-density lipoprotein; LDL, low-density lipoprotein; HbA1c, hemoglobin A1c.

**Supplementary Table 11.** Association between Coronary Artery Disease and %MAP ≥40.3%

|  | Odds ratio (95% Confidence Interval);  *p* value | | | |
| --- | --- | --- | --- | --- |
| Variable | Unadjusted | Model 1 | Model 2 | Model 3 |
| %MAP ≥40.3% (yes/no) | 1.76 (1.38-2.25);  <0.001 | 1.86 (1.43-2.42);  <0.001 | 1.94 (1.45-2.59);  <0.001 | 1.63 (1.19-2.24);  0.002 |
| Age (year) | - | 1.06 (1.05-1.07);  <0.001 | 1.05 (1.04-1.06);  <0.001 | 1.04 (1.03-1.06);  <0.001 |
| Male (yes/no) | - | 4.35 (3.16-5.99);  <0.001 | 3.90 (2.61-5.83);  <0.001 | 3.83 (2.56-5.73);  <0.001 |
| Body mass index (kg/m^2^) | - | - | 0.95 (0.91-0.99);  0.03 | 0.96 (0.92-1.00);  0.06 |
| Heart rate (bpm) | - | - | 0.96 (0.95-0.98);  <0.001 | 0.96 (0.95-0.97);  <0.001 |
| Hypertension (yes/no) | - | - | 1.57 (0.98-2.52);  0.06 | 1.63 (1.01-2.62);  0.04 |
| Dyslipidemia (yes/no) | - | - | 11.9 (6.50-21.9);  <0.001 | 12.6 (6.81-23.3);  <0.001 |
| Diabetes mellitus (yes/no) | - | - | 2.35 (1.77-3.23);  <0.001 | 2.27 (1.70-3.03);  <0.001 |
| Smoking (yes/no) | - | - | 1.91 (1.34-2.74);  <0.001 | 1.86 (1.30-2.66);  <0.001 |
| ABI (×100) | - | - | - | 0.98 (0.97-0.99);  0.002 |

%MAP indicates percentage of mean arterial pressure; ABI, ankle-brachial index.

**Supplementary Figure 1.**
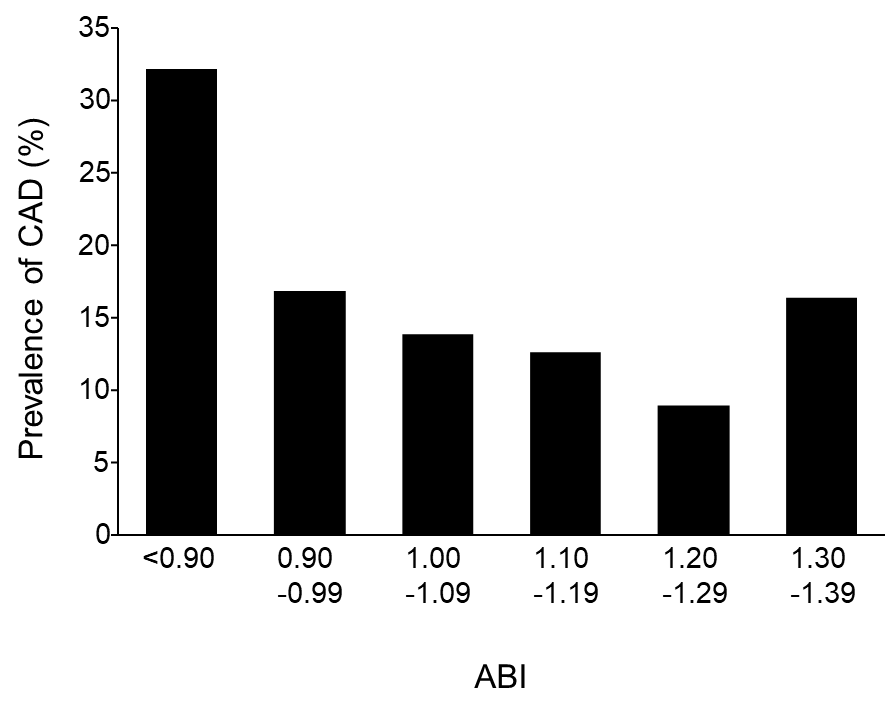


Bar graphs show the prevalence of coronary artery disease (CAD) according to ankle-brachial index (ABI).
